# Synthesis of a new series of 3-functionalised-1-phenyl-1,2,3-triazole sulfamoylbenzamides as carbonic anhydrase I, II, IV and IX inhibitors

**DOI:** 10.1080/14756366.2019.1629432

**Published:** 2019-06-25

**Authors:** Baijayantimala Swain, Andrea Angeli, Srinivas Angapelly, Pavitra S. Thacker, Priti Singh, Claudiu T. Supuran, Mohammed Arifuddin

**Affiliations:** a Department of Medicinal Chemistry, National Institute of Pharmaceutical Education and Research (NIPER), Hyderabad, Telangana, India;; b Neurofarba Department, Sezione di Scienze Farmaceutiche e Nutraceutiche, Università degli Studi di Firenze, Sesto Fiorentino, Italy

**Keywords:** Carbonic anhydrase, click tail approach, 3-sulfamoylbenzamide, isoforms CA I, II, IV, IX

## Abstract

The synthesis of a novel series of 3-functionalised benzenesulfonamides incorporating phenyl-1,2,3-triazole with an amide linker was achieved by using the “click-tail” approach. The new compounds, including the intermediates, were assayed as inhibitors of human carbonic anhydrase (CA, EC 4.2.1.1) isoforms hCA I and II (cytosolic isoforms) and also for hCA IV and IX (transmembrane isoforms) taking acetazolamide as standard drug. Most of these compounds exhibited excellent activity against all these isoforms. hCA I was inhibited with *K_i_*s in the range of 50.8–966.8 nM, while the glaucoma associated hCA II was inhibited with *K_i_*s in the range of 6.5–760.0 nM. Isoform hCA IV was inhibited with *K_i_*s in the range of 65.3–957.5 nM, whereas the tumor associated hypoxia induced hCA IX was inhibited with *K_i_*s in the range of 30.8–815.9 nM. The structure activity relationship study for the 3-functionalised-1-phenyl-1,2,3-triazole sulfamoylbenzamides against these isoforms was also inferred from the results.

## 1. Introduction

**Figure 1.**
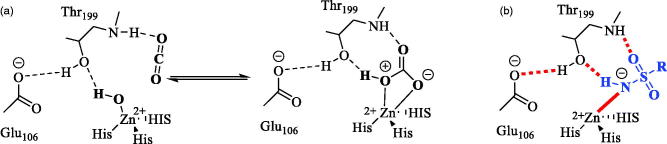
Zinc coordination within CA active site showing: (a) hydration of CO_2_ to HCO_3–_ and (b) a sulfonamide inhibitor bound to the zinc ion and the gate keeping residues Thr199-Glu106, conserved in all α-CAs [4].

Many approaches for the development of sulfonamide carbonic anhydrase (CA, EC 4.2.1.1) inhibitors have been explored with the aim to achieve better selectivity profiles towards the different human (h) isoforms of the enzyme. CAs are ubiquitous Zn containing metalloenzymes present in all life phyla catalyzing the reversible hydration of carbon dioxide to bicarbonate anion and a proton by using a metal hydroxide nucleophilic mechanism and is being crucial for a variety of physiological and pathological processes such as maintenance pH and CO_2_ homeostasis, electrolyte secretion, bone resorption/calcification, gluconeogenesis, cell differentiation and proliferation, neurotransmission (in mammals), and virulence and tissue colonization (in pathogens)^1^. Up until now it has already been reported that sulfonamides, sulfamates, sulfamides, hydroxamates incorporate efficient zinc binding groups (ZBGs) and directly bind to the metal ion within the enzyme cavity^2^. Thus, from the drug design view point Zn^2+^ is the interesting target where the classical sulfonamide group (–SO_2_NH_2_) is the recognition motif for small molecules. In deprotonated form it binds to the zinc (II) ion in the active site thereby inhibiting the binding of the endogenous substrates (CO_2_ and H_2_O) and hence reducing the catalytic ability of the enzyme, similar to the transition state of the endogenous reaction constituting two additional H-bonds with Thr199 residue (Figure 1)^3^^,^^4^. The binding pattern for the sulfonamide moiety exhibits a common feature among the active site super structure of all the 15 human isoenzymes belonging to α-class. The active site consists of a tetrahedral Zn^2+^ coordinated to the imidazole side chains of the three histidine residues present at the base of the funnel shaped active site cavity. Till now seven genetically distinct families have been identified the α, β, γ, δ, ζ, η, θ-CAs, which are very much different from the α-CAs mentioned above^5–7^.

**Figure 2.**
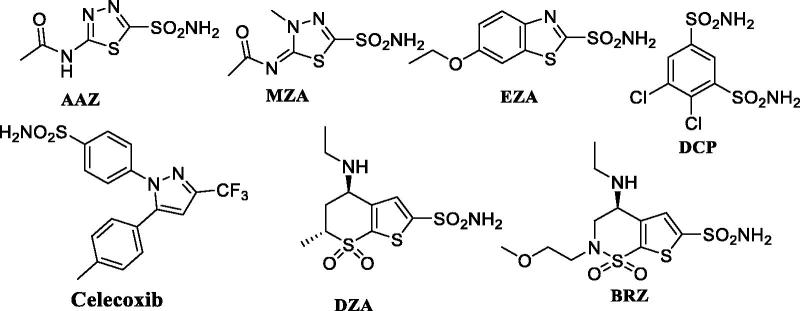
Clinically used classical sulfonamide CA inhibitors.

The hCA isoforms differ in their subcellular location, tissue distribution, and molecular and kinetic properties. Basically, four isoforms are cytosolic (I, II, III, and VII), five membrane-bound (IV, IX, XII, XIV, and XV), two mitochondrial (VA and VB), and CAVI is secreted in saliva and milk. Among these CA IV and XV are having GPI (glycosylphosphatidylinositol) tails anchored to the membrane while CAs IX, XII, XIV are transmembrane proteins possessing just one membrane domain^8^. Despite that, all these five membrane-bound isoforms are commonly termed as extracellular CAs due to having their active sites outside the cell. Many sulfonamide-based drugs (Figure 2) such as acetazolamide (AAZ), methazolamide, ethoxzolamide, dorzolamide, brinzolamide, dichlorophenamide, and celecoxib are used clinically for many years as diuretics, anti-epileptics, anti-glaucoma, or as anti-tumor agents^9^^,^^10^.

Recently many researchers in this field are focusing on isoenzyme selective sulfonamide inhibitors by using two strategies such as the ring and the tail approach (first described by Supuran’s group)^11^. The first one consists in modulating the ring directly linked to the sulfonamide moiety and the later one entails attaching different tails to the aromatic/heterocyclic ring bearing the ZBG (Figure 1). The respective tail moieties of the ligand have the ability to specifically interact with amino acid residues (most variable among various isoenzymes) present at the rim of the active site pocket. Hence, the tail approach is followed mostly. It was also possible to harmonise the physicochemical properties (most crucial for activity) of the CAIs by selecting tails with a diverse chemical nature^12^. Although diverse types of sulfonamide derivatives have already been reported for CA inhibition, it is necessary to explore this class further for better CA inhibitory profiles.

**Figure 3.**
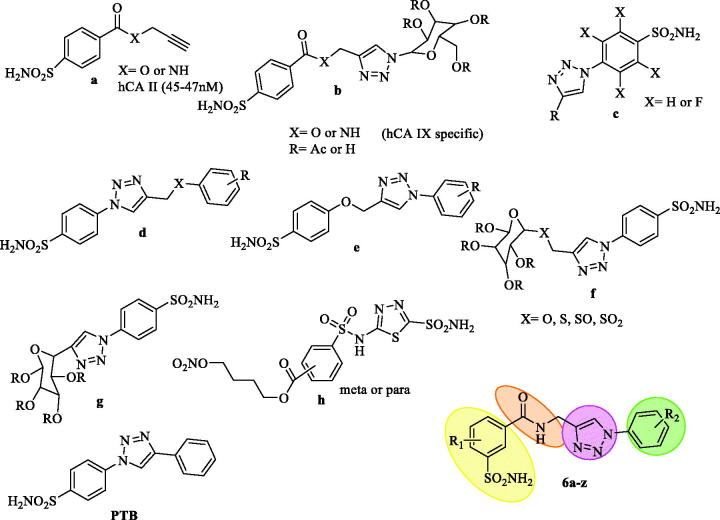
1,2,3-Triazole containing sulfonamide molecules as CAIs based on “Click Tailing” approach.

Nowadays, click chemistry is widely used to obtain metallo-enzyme CA inhibitors belonging to sulfonamide and coumarin classes. To synthesise 1,4-disubstituted 1,2,3-triazoles, the copper-catalyzed azide-alkyne cyclooadditions also well known as “click chemistry” has played a pivotal role in medicinal chemistry. The 1,2,3-triazole ring is a bioisostere of the amide bond and maintains high stability under basic as well as acid hydrolysis, reductive and oxidative conditions. It also has high dipole moment and capability of H-bonding in the *in-viv*o environment. Due to its aromatic character, it may undergo π-stacking interactions with relevant amino acid residues within the enzyme cavity. In recent years, CAIs belonging to sulfonamide and coumarin classes have been obtained by recurrent use of click chemistry. Thus, owing to the versatility of click chemistry in medicinal chemistry and drug discovery, it was combined with the tail approach, together termed as “click tailing” for the first time in 2006, for the development of cell membrane impermeable CA inhibitors. Authors have explored the reversal of CA isoenzyme selectivity from hCA II to hCA IX by tethering a sugar triazole tail on to the CA anchor pharmacophore (Figure 3(a,b))^13^. Pala et al., recently published two series of benzene and tetrafluorobenzenesulfonamide bearing aliphatic or aromatic moieties eliciting inhibition of the cytosolic hCA I and II in medium potencies and the tumor associated CA IX and XII in nanomolar to subnanomolar potencies (Figure 3(c))^14^. Nocentini et al., have synthesised a library of 1,2,3-triazoles endowed with enhanced flexibility compared to the lead PTB (Figure 3(d,e)) and the molecules showed potent inhibition against hCA I, II, IX, XII, and some of them were highly effective for anti-glaucoma activity^15^. Lopez et al., also designed triazoles with and without S-linked glycosyl (Figure 3(f,g)), which are again effective against hCA I, II, IX, and XII isoforms^16^. Recently a novel series of 4-functionalised 1,5-diaryl-1,2,3-triazoles containing benzene sulfonamide moieties described by Pawan et al., showed selectivity towards CA isoforms I, II, IX and XII^17^. All these triazole derivatives above were designed with a 4-sulfamoyl moiety and were having selectivity for the four CA isoforms. The through literature search shows that there is only one report which has a 3-sulfamoyl moiety coupled with 1,3,4-thiadiazole sulfonamide exhibiting exclusively inhibitory action against hCA II (Figure 3(h))^18^. Apart from this report there are no reports on the 3-sulfamoyl substituted derivatives as CAIs.

Thus in the present study we decided to synthesise a novel series of 1,2,3-triazole derivatives linked to a 3-sulfamoyl moiety with an amide linker via the “click tailing” approach and to test them as CA inhibitors.

## 2. Materials and methods

### 2.1. General

All the commercially available reagents were used without further purification. Solvents were dried and distilled wherever necessary prior to use using standard methods. All the air and moisture sensitive reactions were performed under inert conditions using clean and dried glassware and syringe technique to transfer solutions. Reactions were monitored by TLC using Merck silica gel 60 F-254 plates. Purification was performed by column chromatography on silica gel (60–120 mesh) using a mixture of DCM and methanol as eluent. Melting points were acquired on Stuart digital melting point apparatus/SMP 30 in open capillary tubes and uncorrected. Nuclear Magnetic Resonance (^1^H NMR and 13C NMR) spectra were recorded by using an Avance bruker 500 and 125 MHz spectrometer in DMSO-d_6_ as solvent and tetramethylsilane as internal standard. Chemical shifts are reported as δ values in parts per million (ppm) and coupling constants (*J*) are expressed in Hz. Multiplicities are described as s (singlet), d (doublet), t (triplet), q (quartet), m (multiplet), dd (doublet of doublets). HRMS were determined with Agilent QTOF mass spectrometer 6540 series instrument and were performed in the ESI techniques at 70 eV.

### 2.2. Chemistry

#### 2.2.1. Synthesis of 3-(chlorosulfonyl)benzoic acid derivatives (2a–d)

To the stirred chlorosulfonic acid (13.6 ml, 204.5 mmol) at 0 °C, 4-Substituted benzoic acid derivatives (1a–d) (5 g, 40.9 mmol) were added portion wise and then stirred at 110 °C for 5 to 8 h. After completion of the reaction (monitored by TLC) it was cooled to RT and then the reaction mixture is poured into crushed ice (200 g) with vigorous stirring. The solid obtained was filtered off and the residue collected and washed with 50 ml water and dried *in vacuo* to obtain desired intermediate (2a–d) as white solid with 70–85% yield.

#### 2.2.2. Synthesis of 3-(sulfamoyl)benzoic acids (3a–d)

To the ice-cold solution of ammonium hydroxide (25% in water) (5 ml, 122.7 mmol, 30.0 equiv) 3-(chlorosulfonyl)benzoic acid derivatives (2a–d) (0.5 g, 4.09 mmol, 1.0 equiv) were added portion wise and stirred for 2–3 h at room temperature. After the reaction was completed (monitored by TLC) the solvent was removed under reduced pressure. The residue was suspended in 5 ml of water and quenched with 2–5 ml of Conc. HCl. The precipitate obtained was collected by vacuum filtration and was washed with 10 ml of water and dried to obtain **3a–d** as white solid with 85–95% yield.

#### 2.2.3. Synthesis of N-(prop-2-yn-1-yl)-3-sulfamoylbenzamide (4a–d)

To the stirred solution of 3-(sulfamoyl)benzoic acid derivatives **3a–d** (0.5 g, 2.5 mmol) in dry DMF (5 ml), EDCI (2.75 mmol), and HOBt (2.75 mmol) were added under inert conditions and the resultant solution stirred for 30 min at room temperature. This was followed by addition of propagyl amine (2.75 mmol) and the resultant solution was stirred at room temperature until the reaction was completed (monitored by TLC). After completion of the reaction as indicated by TLC, the reaction mixture was quenched with ice and the precipitate obtained is filtered and washed with ice cold water. The crude product was purified by column chromatography using alumina as the stationary phase and DCM: Methanol (97:3) as eluent to afford the products as white solid in 70–80% yield.

#### 2.2.4. Synthesis of N-((1-phenyl-1H-1,2,3-triazol-4-yl)methyl)-3-sulfamoylbenzamides (6a-z) via click chemistry

*N*-(prop-2-yn-1-yl)-3-sulfamoylbenzamides **4a–d** (0.08 g, 0.34 mmol) and phenyl azides (**5a–m)** (0.37 mmol) were dissolved in *^t^*BuOH/H_2_O (1:1, 5 ml) followed by the addition of CuSO_4_.5H_2_O (0.07 mmol) and sodium ascorbate (0.14 mmol). The resultant solution was kept for stirring till completion of the reaction (TLC monitoring). Solvents were removed under vacuum and the residue was purified by column chromatography using silica gel (60–120 mesh) as the stationary phase and methanol in DCM (0–5%) as the mobile phase. The pure products (**6a–z)** were collected in 52–98% yield.

##### 2.2.4.1. 3-Sulfamoylbenzoic acid (3a):

White solid, Yield 95%; ^1^H NMR (500 MHz, DMSO) δ 13.42 (s, 1H), 8.40 (t, *J* = 1.7 Hz, 1H), 8.15 (dd, *J* = 7.7, 1.1 Hz, 1H), 8.06 (dd, *J* = 7.9, 1.3 Hz, 1H), 7.72 (dd, *J* = 9.7, 5.8 Hz, 1H), 7.51 (s, 2H). 13C NMR (125 MHz, DMSO) δ 166.67, 145.09, 132.83, 132.00, 130.17, 130.07, 126.91.

##### 2.2.4.2. 4-Chloro-3-sulfamoylbenzoic acid (3b)

White solid, Yield 85%; ^1^H NMR (500 MHz, DMSO) δ 13.44 (s, 1H), 8.36 (dt, *J* = 10.0, 5.0 Hz, 1H), 8.23–8.17 (m, 1H), 7.86 (s, 2H), 7.56 (dt, *J* = 14.7, 7.4 Hz, 1H). 13C NMR (125 MHz, DMSO) δ 165.91, 136.02 (d, *J* = 9.9 Hz), 132.34 (d, *J* = 15.4 Hz), 130.21, 127.78 (d, *J* = 3.4 Hz), 118.32, 118.22 (d, *J* = 22.1 Hz).

##### 2.2.4.3. 4-Fluoro-3-sulfamoylbenzoic acid (3c)

White solid, Yield 87%; ^1^H NMR (500 MHz, DMSO) δ 13.46 (s, 1H), 8.39–8.32 (m, 1H), 8.23–8.15 (m, 1H), 7.88 (s, 2H), 7.56 (dt, *J* = 15.4, 7.7 Hz, 1H). 13C NMR (125 MHz, DMSO) δ 165.90, 160.10, 136.04, 135.97, 132.40, 132.28, 130.21, 127.79, 118.30, 118.13.

##### 2.2.4.4. 4-Methoxy-3-sulfamoylbenzoic acid (3d)

White solid, Yield 92%; ^1^H NMR (500 MHz, DMSO) δ 12.94 (s, 1H), 8.32 (t, *J* = 3.1 Hz, 1H), 8.17–8.08 (m, 1H), 7.32 (d, *J* = 8.7 Hz, 1H), 7.23 (s, 2H), 3.99 (s, 3H). 13C NMR (125 MHz, DMSO) δ 166.62, 159.85, 135.49, 131.74, 129.54, 122.79, 113.20, 57.07. HRMS (ESI) *m*/*z*: [M + Na]^+^ calculated for C_8_H_9_NNaO_5_S 254.0099, found 254.0098.

##### 2.2.4.5. N-(prop-2-yn-1-yl)-3-sulfamoylbenzamide (4a)

White solid, Yield 80%; ^1^H NMR (500 MHz, DMSO) δ 9.19 (t, *J* = 5.4 Hz, 1H), 8.33 (t, *J* = 1.7 Hz, 1H), 8.10–8.03 (m, 1H), 8.01–7.96 (m, 1H), 7.69 (dd, *J* = 14.2, 6.4 Hz, 1H), 7.45 (s, 2H), 4.09 (dd, *J* = 5.5, 2.5 Hz, 2H), 3.15 (t, *J* = 2.5 Hz, 1H). 13C NMR (125 MHz, DMSO) δ 165.31, 144.96, 135.00, 130.68, 129.71, 128.85, 125.32, 81.50, 73.49, 29.14. HRMS (ESI) *m*/*z*: [M + Na]^+^ calculated for C_10_H_10_N_2_NaO_3_S 261.0310, found 261.0310.

##### 2.2.4.6. 4-Chloro-N-(prop-2-yn-1-yl)-3-sulfamoylbenzamide (4b)

White solid, Yield 76%; ^1^H NMR (500 MHz, DMSO) δ 9.26 (t, *J* = 5.4 Hz, 1H), 8.48 (dd, *J* = 5.4, 2.1 Hz, 1H), 8.05 (dd, *J* = 8.2, 2.1 Hz, 1H), 7.78 (t, *J* = 6.1 Hz, 1H), 7.72 (s, 2H), 4.07 (ddd, *J* = 12.3, 5.5, 2.4 Hz, 2H), 3.16 (t, *J* = 2.4 Hz, 1H). 13C NMR (125 MHz, DMSO) δ 164.51, 141.67, 133.92, 133.24, 132.21, 132.00, 128.68, 81.37, 73.62, 29.19. HRMS (ESI) *m*/*z*: [M + H]^+^ calculated for C_10_H_10_ClN_2_O_3_S^+^ 273.0095, found 273.0010.

##### 2.2.4.7. 4-Fluoro-N-(prop-2-yn-1-yl)-3-sulfamoylbenzamide (4c)

White solid, Yield 70%; ^1^H NMR (500 MHz, DMSO) δ 9.21 (t, *J* = 5.4 Hz, 1H), 8.33 (dd, *J* = 7.0, 2.2 Hz, 1H), 8.14 (ddd, *J* = 8.5, 4.5, 2.3 Hz, 1H), 7.77 (s, 2H), 7.56 (t, *J* = 9.2 Hz, 1H), 4.08 (dd, *J* = 5.4, 2.5 Hz, 2H), 3.21–3.09 (m, 1H). 13C NMR (125 MHz, DMSO) δ 164.39, 159.20, 133.79, 133.72, 132.21, 132.09, 130.65, 128.58, 117.85, 117.67, 81.44, 73.54, 73.50, 29.18. HRMS (ESI) *m*/*z*: [M + H]^+^ calculated for C_10_H_10_FN_2_O_3_S^+^ 257.0391, found 257.0397.

##### 2.2.4.8. 4-Methoxy-N-(prop-2-yn-1-yl)-3-sulfamoylbenzamide (4d)

White solid, Yield 79%; ^1^H NMR (500 MHz, DMSO) δ 9.03 (t, *J* = 5.4 Hz, 1H), 8.31 (dd, *J* = 12.1, 2.2 Hz, 1H), 8.19–7.98 (m, 1H), 7.37–7.27 (m, 1H), 7.17 (s, 2H), 4.09–4.02 (m, 2H), 3.97 (d, *J* = 3.7 Hz, 3H), 3.20–3.07 (m, 1H). 13C NMR (125 MHz, DMSO) δ 164.96, 158.75, 133.17, 131.60, 127.80, 125.72, 112.87, 81.77, 73.33, 56.97, 29.02. HRMS (ESI) *m*/*z*: [M + H]^+^ calculated for C_11_H_13_N_2_O_4_S^+^ 269.0591, found 269.0591.

##### 2.2.4.9. N-((1-phenyl-1H-1,2,3-triazol-4-yl)methyl)-3-sulfamoylbenzamide (6a)

White solid; yield: 98%, m.p: 214–216 °C; ^1^H NMR (500 MHz, DMSO) δ 9.32 (t, *J* = 5.4 Hz, 1H), 8.70 (s, 1H), 8.37 (s, 1H), 8.12 (d, *J* = 7.8 Hz, 1H), 7.98 (d, *J* = 7.8 Hz, 1H), 7.91 (d, *J* = 7.7 Hz, 2H), 7.68 (dd, *J* = 17.5, 9.8 Hz, 1H), 7.59 (t, *J* = 7.9 Hz, 2H), 7.47 (dd, *J* = 14.1, 6.7 Hz, 1H), 7.44 (s, 2H), 4.64 (d, *J* = 5.5 Hz, 2H).13C NMR (125 MHz, DMSO) δ 165.56, 146.40, 144.92, 137.17, 135.32, 130.78, 130.34, 129.58, 129.03, 128.71, 125.38, 121.71, 120.46, 35.45. HRMS (ESI) *m*/*z*: [M + H]^+^ calculated for C_16_H_16_N_5_O_3_S 358.0968, found 358.0976.

#### 2.2.5.0. 3-Sulfamoyl-N-((1–(3,4,5-trimethoxyphenyl)-1H-1,2,3-triazol-4-yl)methyl)benzamide (6b)

White solid; yield: 98%, m.p: 230–231 °C; ^1^H NMR (500 MHz, DMSO) δ 9.34 (t, *J* = 5.6 Hz, 1H), 8.73 (s, 1H), 8.37 (s, 1H), 8.11 (d, *J* = 7.8 Hz, 1H), 7.98 (d, *J* = 7.8 Hz, 1H), 7.69 (t, *J* = 7.8 Hz, 1H), 7.44 (s, 2H), 7.20 (s, 2H), 4.64 (d, *J* = 5.6 Hz, 2H), 3.87 (s, 6H), 3.71 (s, 3H).13C NMR(125 MHz, DMSO) δ 165.49, 154.00, 146.19, 144.94, 137.85, 135.28, 133.06, 130.76, 129.61, 128.74, 125.39, 98.56, 60.67, 56.81, 35.41. HRMS (ESI) *m*/*z*: [M + H]^+^ calculated for C_19_H_22_N_5_O_6_S 448.1285, found 448.1293.

##### 2.2.5.1. N-((1–(3-nitrophenyl)-1H-1,2,3-triazol-4-yl)methyl)-3-sulfamoylbenzamide (6c)

White solid; yield: 92%, m.p: 212–213 °C; ^1^H NMR (500 MHz, DMSO) δ 9.36 (t, *J* = 5.6 Hz, 1H), 8.97 (s, 1H), 8.74 (t, *J* = 2.1 Hz, 1H), 8.44–8.40 (m, 1H), 8.37 (t, *J* = 1.6 Hz, 1H), 8.34–8.29 (m, 1H), 8.14–8.10 (m, 1H), 8.00–7.96 (m, 1H), 7.89 (t, *J* = 8.2 Hz, 1H), 7.70 (t, *J* = 7.8 Hz, 1H), 7.44 (s, 2H), 4.66 (d, *J* = 5.6 Hz, 2H).13C NMR (125 MHz, DMSO) δ 165.57, 149.07, 147.01, 144.95, 137.73, 135.26, 132.00, 130.77, 129.60, 128.76, 126.46, 125.39, 123.47, 122.20, 115.13, 35.42. HRMS (ESI) *m*/*z*: [M + H]^+^ calculated for C_16_H_15_N_6_O_5_S 403.0819, found 403.0826.

##### 2.2.5.2. N-((1–(3-fluorophenyl)-1H-1,2,3-triazol-4-yl)methyl)-3-sulfamoylbenzamide (6d)

White solid, yield: 96%, m.p: 209–210 °C; ^1^H NMR (500 MHz, DMSO) δ 9.34 (t, *J* = 5.5 Hz, 1H), 8.77 (s, 1H), 8.37 (t, *J* = 1.5 Hz, 1H), 8.11 (d, *J* = 7.9 Hz, 1H), 7.98 (d, *J* = 8.4 Hz, 1H), 7.88–7.78 (m, 2H), 7.72–7.59 (m, 2H), 7.44 (s, 2H), 7.33 (tt, *J* = 10.9, 5.5 Hz, 1H), 4.64 (d, *J* = 5.6 Hz, 2H).13C NMR (125 MHz, DMSO) δ 165.56, 146.66, 144.93, 135.28, 132.29 (d, *J* = 9.1 Hz), 130.77, 129.59, 128.74, 125.38, 121.88, 116.32 (d, *J* = 2.9 Hz), 115.81, 115.64, 107.99, 107.78, 35.41. HRMS (ESI) *m*/*z*: [M + H]^+^ calculated for C_16_H_15_N_5_O_3_S 376.0874, found 376.0883.

##### 2.2.5.3. N-((1–(3-cyanophenyl)-1H-1,2,3-triazol-4-yl)methyl)-3-sulfamoylbenzamide (6e)

White solid, yield: 87%, m.p: 238–240 °C; ^1^H NMR (500 MHz, DMSO) δ 9.37 (t, *J* = 5.6 Hz, 1H), 8.83 (s, 1H), 8.49–8.43 (m, 1H), 8.37 (t, *J* = 1.6 Hz, 1H), 8.34–8.28 (m, 1H), 8.12 (d, *J* = 7.9 Hz, 1H), 7.97 (dd, *J* = 17.3, 7.8 Hz, 2H), 7.80 (t, *J* = 8.0 Hz, 1H), 7.69 (dd, *J* = 17.6, 9.8 Hz, 1H), 7.50–7.40 (m, 2H), 4.65 (d, *J* = 5.6 Hz, 2H).13C NMR (125 MHz, DMSO) δ 165.57, 146.94, 144.95, 137.56, 135.25, 132.61, 131.75, 130.76, 129.60, 125.38, 125.02, 123.75, 121.92, 118.27 113.29, 35.42. HRMS (ESI) *m*/*z*: [M + H]^+^ calculated for C_17_H_15_N_6_O_3_S 383.0921, found 383.0928.

##### 2.2.5.4. N-((1–(4-methoxyphenyl)-1H-1,2,3-triazol-4-yl)methyl)-3-sulfamoylbenzamide (6f)

White solid, yield: 91%, m.p: 219–220 °C; ^1^H NMR (500 MHz, DMSO) δ 9.31 (t, *J* = 5.6 Hz, 1H), 8.35 (t, *J* = 1.6 Hz, 1H), 8.32 (s, 1H), 8.11–8.08 (m, 1H), 7.98–7.95 (m, 1H), 7.68 (t, *J* = 7.8 Hz, 1H), 7.60 (dd, *J* = 7.8, 1.6 Hz, 1H), 7.54–7.50 (m, 1H), 7.43 (s, 2H), 7.31 (dd, *J* = 8.4, 0.8 Hz, 1H), 7.13 (td, *J* = 7.7, 1.1 Hz, 1H), 4.64 (d, *J* = 5.6 Hz, 2H), 3.85 (s, 3H).13C NMR (125 MHz, DMSO) δ 165.59, 152.05, 144.96 (d, *J* = 12.8 Hz), 135.39, 131.08, 130.76, 129.59, 128.69, 126.13, 125.35, 121.36, 113.55, 56.61, 35.36. HRMS (ESI) *m*/*z*: [M + H]^+^ calculated for C_17_H_18_N_5_O_4_S 388.1074, found 388.1082.

##### 2.2.5.5. N-((1–(2,3-dimethylphenyl)-1H-1,2,3-triazol-4-yl)methyl)-3-sulfamoylbenzamide (6g)

White solid; yield: 86%, m.p: 205–206 °C; ^1^H NMR (500 MHz, DMSO) δ 9.30 (t, *J* = 5.5 Hz, 1H), 8.36 (s, 1H), 8.27 (s, 1H), 8.09 (t, *J* = 9.4 Hz, 1H), 7.97 (d, *J* = 7.8 Hz, 1H), 7.68 (t, *J* = 7.8 Hz, 1H), 7.44 (s, 2H), 7.39 (d, *J* = 7.6 Hz, 1H), 7.28 (t, *J* = 7.7 Hz, 1H), 7.20 (d, *J* = 7.8 Hz, 1H), 4.65 (d, *J* = 5.5 Hz, 2H), 2.34 (s, 3H), 1.96 (s, 3H).13C NMR (125 MHz, DMSO) δ 165.59, 145.21, 144.91, 138.91, 136.95, 135.40, 132.64, 131.48, 130.78, 129.57, 128.69, 126.62, 125.37, 124.36, 35.42, 20.34, 14.40. HRMS (ESI) *m*/*z*: [M + H]^+^ calculated for C_18_H_20_N_5_O_3_S 386.1281, found 386.1287.

##### 2.2.5.6. N-((1–(2,4-dimethylphenyl)-1H-1,2,3-triazol-4-yl)methyl)-3-sulfamoylbenzamide (6h)

White solid, yield: 89%, m.p: 189–191 °C; ^1^H NMR (500 MHz, DMSO) δ 9.29 (t, *J* = 5.4 Hz, 1H), 8.35 (d, *J* = 1.5 Hz, 1H), 8.29 (s, 1H), 8.10 (d, *J* = 7.8 Hz, 1H), 7.97 (d, *J* = 8.3 Hz, 1H), 7.68 (t, *J* = 7.8 Hz, 1H), 7.43 (s, 2H), 7.27 (d, *J* = 7.7 Hz, 2H), 7.19 (d, *J* = 8.0 Hz, 1H), 4.64 (d, *J* = 5.5 Hz, 2H), 2.36 (s, 3H), 2.11 (s, 3H). 13C NMR (125 MHz, DMSO) δ 165.56, 145.17, 144.91, 139.78, 135.39, 134.46, 133.12, 132.23, 130.78, 129.57, 128.69, 127.83, 126.18, 125.37, 125.06, 35.41, 21.07, 17.82. HRMS (ESI) *m*/*z*: [M + H]^+^ calculated for C_18_H_20_N_5_O_3_S 386.1281, found 386.1288.

##### 2.2.5.7. 4-Chloro-N-((1-phenyl-1H-1,2,3-triazol-4-yl)methyl)-3-sulfamoylbenzamide (6i)

White solid, yield: 83%, m.p: 199–201 °C; ^1^H NMR (500 MHz, DMSO) δ 9.38 (t, *J* = 5.5 Hz, 1H), 8.70 (s, 1H), 8.51 (d, *J* = 2.1 Hz, 1H), 8.10 (dd, *J* = 8.3, 2.2 Hz, 1H), 7.90 (dd, *J* = 8.5, 0.9 Hz, 2H), 7.76 (t, *J* = 10.0 Hz, 1H), 7.69 (s, 2H), 7.59 (dd, *J* = 10.8, 5.0 Hz, 2H), 7.47 (dd, *J* = 17.5, 10.1 Hz, 1H), 4.63 (d, *J* = 5.5 Hz, 2H).13C NMR (125 MHz, DMSO) δ 164.77, 146.28, 141.63, 137.16, 133.68 (d, *J* = 19.8 Hz), 132.08, 130.34, 129.04, 128.79, 121.73, 120.45, 35.49. HRMS (ESI) *m*/*z*: [M + H]^+^ calculated for C_16_H_15_ClN_5_O_3_S 392.0579, found 392.0586.

##### 2.2.5.8. 4-Chloro-3-sulfamoyl-N-((1–(3,4,5-trimethoxyphenyl)-1H-1,2,3-triazol-4-yl)methyl)-benzamide (6j)

White solid, yield: 95%, m.p: 249–251 °C; ^1^H NMR (500 MHz, DMSO) δ 9.41 (t, *J* = 5.5 Hz, 1H), 8.73 (s, 1H), 8.51 (d, *J* = 2.0 Hz, 1H), 8.10 (dd, *J* = 8.3, 2.1 Hz, 1H), 7.77 (d, *J* = 8.3 Hz, 1H), 7.69 (s, 2H), 7.20 (s, 2H), 4.63 (d, *J* = 5.5 Hz, 2H), 3.87(s, 6H), 3.71 (s, 3H).13C NMR (125 MHz, DMSO) δ 164.72, 154.01, 146.06, 141.66, 137.89, 133.80, 133.57, 133.06, 132.09 (d, *J* = 4.4 Hz), 128.79, 122.05, 98.59, 60.68, 56.82, 35.45. HRMS (ESI) *m*/*z*: [M + H]^+^ calculated for C_19_H_21_ClN_5_O_6_S 482.0896, found 482.0899.

##### 2.2.5.9. 4-Chloro-N-((1–(4-methoxyphenyl)-1H-1,2,3-triazol-4-yl)methyl)-3-sulfamoylbenzamide(6k)

White solid, yield: 90%, m.p: 172–174 °C; ^1^H NMR (500 MHz, DMSO) δ 9.37 (t, *J* = 5.5 Hz, 1H), 8.59 (s, 1H), 8.51 (d, *J* = 2.1 Hz, 1H), 8.09 (dd, *J* = 8.3, 2.2 Hz, 1H), 7.82–7.75 (m, 3H), 7.69 (s, 2H), 7.15–7.08 (m, 2H), 4.61 (d, *J* = 5.5 Hz, 2H), 3.82 (s, 3H).13C NMR (125 MHz, DMSO) δ 164.76, 159.69, 146.01, 141.64, 133.69 (d, *J* = 14.6 Hz), 132.07, 130.62, 128.79, 122.11, 121.71, 115.35, 56.04, 35.50. HRMS (ESI) *m*/*z*: [M + H]^+^ calculated for C_17_H_17_ClN_5_O_4_S 422.0684, found 422.0690.

#### 2.2.6. 4-Chloro-N-((1–(4-fluorophenyl)-1H-1,2,3-triazol-4-yl)methyl)-3-sulfamoylbenzamide(6l)

White solid, yield: 83%, m.p: 189–190 °C; ^1^H NMR (500 MHz, DMSO) δ 9.39 (t, *J* = 5.6 Hz, 1H), 8.69 (s, 1H), 8.51 (d, *J* = 2.2 Hz, 1H), 8.10 (dd, *J* = 8.3, 2.2 Hz, 1H), 7.98–7.91 (m, 2H), 7.78 (d, *J* = 8.3 Hz, 1H), 7.70 (s, 2H), 7.47–7.39 (m, 2H), 4.62 (d, *J* = 5.6 Hz, 2H).13C NMR (125 MHz, DMSO) δ 164.76, 163.03, 146.35, 141.64, 133.76, 133.58, 132.07, 128.78, 122.79 (d, *J* = 8.8 Hz), 121.98, 117.25, 117.07, 35.46. HRMS (ESI) *m*/*z*: [M + H]^+^ calculated for C_16_H_14_ ClFN_5_O_3_S 410.0484, found 410.0492.

##### 2.2.6.1. 4-Chloro-3-sulfamoyl-N-((1–(4-(trifluoromethyl)phenyl)-1H-1,2,3-triazol-4-yl)methyl)benzamide(6m)

White solid, yield: 92%, m.p: 178–180 °C; ^1^H NMR (500 MHz, DMSO) δ 9.42 (t, *J* = 5.6 Hz, 1H), 8.87 (s, 1H), 8.52 (d, *J* = 2.1 Hz, 1H), 8.18 (d, *J* = 8.5 Hz, 2H), 8.10 (dd, *J* = 8.3, 2.2 Hz, 1H), 8.03–7.92 (m, 2H), 7.82–7.74 (m, 1H), 7.70 (s, 2H), 4.64 (d, *J* = 5.5 Hz, 2H).13C NMR (125 MHz, DMSO) δ 164.79, 146.78, 141.65, 139.91, 133.80, 133.54, 132.08, 128.78, 127.64, 122.00, 120.89, 35.44. HRMS (ESI) *m*/*z*: [M + H]^+^ calculated for C_17_H_14_ClF_3_N_5_O_3_S 460.0452, found 460.0343.

##### 2.2.6.2. 4-Fluoro-N-((1-phenyl-1H-1,2,3-triazol-4-yl)methyl)-3-sulfamoylbenzamide (6n)

White solid, yield: 87%, m.p: 192–194 °C; ^1^H NMR (500 MHz, DMSO) δ 9.35 (t, *J* = 5.3 Hz, 1H), 8.70 (s, 1H), 8.37 (dd, *J* = 6.9, 2.0 Hz, 1H), 8.24–8.14 (m, 1H), 7.90 (d, *J* = 7.8 Hz, 2H), 7.76 (s, 2H), 7.64–7.52 (m, 3H), 7.48 (t, *J* = 7.4 Hz, 1H), 4.63 (d, *J* = 5.5 Hz, 2H).13C NMR (125 MHz, DMSO) δ 164.65, 146.36, 137.16, 133.81 (d, *J* = 9.6 Hz), 132.11 (d, *J* = 15.0 Hz), 131.00, 130.34, 129.03, 128.65, 121.71, 120.45, 117.97–117.71 (m), 117.60 (d, *J* = 22.3 Hz), 35.48. HRMS (ESI) *m*/*z*: [M + H]^+^ calculated for C_16_H_15_FN_5_O_3_S 376.0874, found 376.0881.

##### 2.2.6.3. 4-Fluoro-N-((1–(4-methoxyphenyl)-1H-1,2,3-triazol-4-yl)methyl)-3-sulfamoylbenzamide (6o)

White solid, yield: 85%,m.p: 209–211 °C; ^1^H NMR (500 MHz, DMSO) δ 9.33 (t, *J* = 5.4 Hz, 1H), 8.59 (s, 1H), 8.37 (dd, *J* = 7.0, 2.2 Hz, 1H), 8.18 (ddd, *J* = 8.4, 4.5, 2.3 Hz, 1H), 7.83–7.78 (m, 2H), 7.76 (s, 2H), 7.59–7.51 (m, 1H), 7.16–7.08 (m, 2H), 4.61 (d, *J* = 5.5 Hz, 2H), 3.82 (s, 3H).13C NMR (125 MHz, DMSO) δ 164.63, 159.67, 146.08, 133.81 (d, *J* = 9.3 Hz), 132.10 (d, *J* = 15.0 Hz), 130.99 (d, *J* = 3.5 Hz), 130.61, 128.64, 122.10, 121.68, 117.69, 117.52, 115.34, 56.03, 35.48. HRMS (ESI) *m*/*z*: [M + H]^+^ calculated for C_17_H_17_FN_5_O_4_S 406.0980, found 406.0988.

##### 2.2.6.4. 4-Fluoro-3-sulfamoyl-N-((1–(4-(trifluoromethyl)phenyl)-1H-1,2,3-triazol-4-yl)methyl)benzamide (6p)

White solid, yield: 79%, m.p: 157–158 °C; ^1^H NMR (500 MHz, DMSO) δ 9.38 (t, *J* = 5.6 Hz, 1H), 8.87 (s, 1H), 8.37 (dd, *J* = 7.0, 2.3 Hz, 1H), 8.23–8.14 (m, 3H), 7.96 (t, *J* = 9.9 Hz, 2H), 7.76 (s, 2H), 7.55 (dt, *J* = 18.9, 9.5 Hz, 1H), 4.64 (d, *J* = 5.6 Hz, 2H). 13C NMR (125 MHz, DMSO) δ 164.67, 146.85, 139.91, 133.81 (d, *J* = 9.3 Hz), 132.13 (d, *J* = 15.0 Hz), 130.92 (d, *J* = 3.4 Hz), 129.20, 128.94, 128.64, 127.65 (d, *J* = 3.9 Hz), 121.98, 120.89, 117.94–117.77 (m), 117.63 (d, *J* = 22.1 Hz), 35.43. HRMS (ESI) *m*/*z*: [M + H]^+^ calculated for C_17_H_14_F_4_N_5_O_3_S 444.0748, found 444.0757.

##### 2.2.6.5. 4-Fluoro-N-((1–(3-fluorophenyl)-1H-1,2,3-triazol-4-yl)methyl)-3-sulfamoylbenzamide (6q)

White solid, yield: 76%, m.p: 230–232 °C; ^1^H NMR (500 MHz, DMSO) δ 9.36 (t, *J* = 5.5 Hz, 1H), 8.77 (s, 1H), 8.37 (dd, *J* = 7.0, 2.2 Hz, 1H), 8.19 (ddd, *J* = 8.5, 4.5, 2.3 Hz, 1H), 7.88–7.78 (m, 2H), 7.76 (s, 2H), 7.68–7.60 (m, 1H), 7.58–7.52 (m, 1H), 7.33 (td, *J* = 8.4, 2.2 Hz, 1H), 4.62 (d, *J* = 5.6 Hz, 2H).13C NMR (125 MHz, DMSO) δ 164.65, 146.62, 138.38 (d, *J* = 10.7 Hz), 133.81 (d, *J* = 9.4 Hz), 132.29 (d, *J* = 9.3 Hz), 130.94 (d, *J* = 3.4 Hz), 128.64, 121.88, 118.07–117.72 (m), 117.62 (d, *J* = 22.1 Hz), 116.32 (d, *J* = 3.0 Hz), 115.81, 115.64, 107.99, 107.78, 35.44. HRMS (ESI) *m*/*z*: [M + H]^+^ calculated for C_16_H_14_F_2_N_5_O_3_S 394.0780, found 394.0786.

##### 2.2.6.6. 4-Fluoro-3-sulfamoyl-N-((1-(p-tolyl)-1H-1,2,3-triazol-4-yl)methyl)benzamide (6r)

White solid, yield: 71%, m.p: 175–177 °C; ^1^H NMR (500 MHz, DMSO) δ 9.34 (t, *J* = 5.5 Hz, 1H), 8.65 (s, 1H), 8.37 (dd, *J* = 7.0, 2.2 Hz, 1H), 8.19 (ddd, *J* = 8.5, 4.5, 2.3 Hz, 1H), 7.82–7.69 (m, 4H), 7.57 (dd, *J* = 22.9, 13.2 Hz, 1H), 7.38 (d, *J* = 8.2 Hz, 2H), 4.61 (d, *J* = 5.5 Hz, 2H), 2.37 (s, 3H).13C NMR (125 MHz, DMSO) δ 164.63, 146.22, 138.63, 134.93, 133.81 (d, *J* = 9.3 Hz), 132.11 (d, *J* = 15.2 Hz), 130.98 (d, *J* = 3.4 Hz), 130.68, 128.65, 121.58, 120.32, 117.69, 117.51, 35.48, 21.01. HRMS (ESI) *m*/*z*: [M + H]^+^ calculated for C_17_H_17_FN_5_O_3_S 390.1031, found 390.1037.

##### 2.2.6.7. N-((1–(3-cyanophenyl)-1H-1,2,3-triazol-4-yl)methyl)-4-fluoro-3-sulfamoylbenzamide (6s)

White solid, yield: 69%, m.p: 249–251 °C; ^1^H NMR (500 MHz, DMSO) δ 9.40 (t, *J* = 5.6 Hz, 1H), 8.82 (s, 1H), 8.48–8.43 (m, 1H), 8.38 (dd, *J* = 7.0, 2.2 Hz, 1H), 8.30 (ddd, *J* = 8.3, 2.2, 0.9 Hz, 1H), 8.19 (ddd, *J* = 8.5, 4.5, 2.3 Hz, 1H), 7.97–7.92 (m, 1H), 7.79 (dd, *J* = 13.4, 5.3 Hz, 1H), 7.77 (s, 2H), 7.57 (dd, *J* = 18.6, 8.9 Hz, 1H), 4.63 (d, *J* = 5.6 Hz, 2H).13C NMR (125 MHz, DMSO) δ 164.67, 146.91, 137.55, 133.79 (d, *J* = 9.4 Hz), 132.61, 131.75, 128.65, 125.02, 123.75, 121.92, 118.27, 117.64 (d, *J* = 21.9 Hz), 117.46–117.19 (m), 116.12–115.96 (m), 113.29, 35.45. HRMS (ESI) *m*/*z*: [M + H]^+^ calculated for C_17_H_14_FN_6_O_3_S 401.0827, found 401.0833.

##### 2.2.6.8. N-((1–(4-bromophenyl)-1H-1,2,3-triazol-4-yl)methyl)-4-fluoro-3-sulfamoylbenzamide (6t)

White solid, yield: 62%, m.p: 153–155 °C; ^1^H NMR (500 MHz, DMSO) δ 9.36 (t, *J* = 5.4 Hz, 1H), 8.74 (s, 1H), 8.37 (dd, *J* = 6.9, 1.9 Hz, 1H), 8.22 – 8.15 (m, 1H), 7.89 (d, *J* = 8.8 Hz, 2H), 7.79 (d, *J* = 8.8 Hz, 2H), 7.76 (s, 2H), 7.56 (t, *J* = 9.2 Hz, 1H), 4.59 (t, *J* = 19.7 Hz, 2H).13C NMR (125 MHz, DMSO) δ 164.65, 146.61, 136.35, 133.80 (d, *J* = 9.3 Hz), 133.23, 132.12 (d, *J* = 15.0 Hz), 130.94 (d, *J* = 3.4 Hz), 128.64, 122.36, 121.70 (d, *J* = 13.7 Hz), 117.70, 117.53, 35.45. HRMS (ESI) *m*/*z*: [M + 2]^+^ calculated for C_17_H_13_BrFN_5_O_3_S 453.9979, found 455.9962.

##### 2.2.6.9. 4-Methoxy-N-((1-phenyl-1H-1,2,3-triazol-4-yl)methyl)-3-sulfamoylbenzamide (6u)

White solid, yield: 58%, m.p: 210–212 °C; ^1^H NMR (500 MHz, DMSO) δ 9.18 (t, *J* = 5.6 Hz, 1H), 8.68 (s, 1H), 8.34 (d, *J* = 2.3 Hz, 1H), 8.14 (dd, *J* = 8.7, 2.3 Hz, 1H), 7.90 (dd, *J* = 8.5, 1.0 Hz, 2H), 7.58 (dd, *J* = 7.9 Hz, 2H), 7.48 (t, *J* = 7.4 Hz, 1H), 7.29 (d, *J* = 8.8 Hz, 1H), 7.16 (s, 2H), 4.61 (d, *J* = 5.5 Hz, 2H), 3.98 (s, 3H).13C NMR (125 MHz, DMSO) δ 165.24, 158.65, 146.63, 137.18, 133.22, 131.61, 130.33, 129.00, 127.88, 126.09, 121.66, 120.44, 112.76, 56.97, 35.37. HRMS (ESI) *m*/*z*: [M + H]^+^ calculated for C_17_H_18_N_5_O_4_S 388.1074, found 388.1079.

#### 2.2.7. N-((1–(4-fluorophenyl)-1H-1,2,3-triazol-4-yl)methyl)-4-methoxy-3-sulfamoylbenzamide (6v)

White solid, yield: 52%, m.p: 222–224 °C; ^1^H NMR (500 MHz, DMSO) δ 9.18 (t, *J* = 5.5 Hz, 1H), 8.66 (s, 1H), 8.33 (d, *J* = 2.1 Hz, 1H), 8.13 (dd, *J* = 8.7, 2.1 Hz, 1H), 8.01–7.85 (m, 2H), 7.48–7.37 (m, 2H), 7.29 (d, *J* = 8.7 Hz, 1H), 7.16 (s, 2H), 4.60 (d, *J* = 5.5 Hz, 2H), 3.96 (s, 3H).13C NMR (125 MHz, DMSO) δ 165.24, 158.68, 146.70, 133.73 (d, *J* = 2.8 Hz), 133.22, 131.61, 127.87, 126.07, 122.79 (d, *J* = 8.7 Hz), 121.92, 117.23, 117.05, 112.77, 56.97, 35.34. HRMS (ESI) *m*/*z*: [M + H]^+^ calculated for C_17_H_17_FN_5_O_4_S 406.0980, found 406.0987.

##### 2.2.7.1. 4-Methoxy-3-sulfamoyl-N-((1-(p-tolyl)-1H-1,2,3-triazol-4-yl)methyl)benzamide (6w)

White solid, yield: 53%, m.p: 211–213 °C; ^1^H NMR (500 MHz, DMSO) δ 9.16 (t, *J* = 5.5 Hz, 1H), 8.62 (s, 1H), 8.33 (d, *J* = 2.0 Hz, 1H), 8.13 (dd, *J* = 8.6, 2.0 Hz, 1H), 7.78 (d, *J* = 8.3 Hz, 2H), 7.38 (d, *J* = 8.4 Hz, 2H), 7.29 (d, *J* = 8.7 Hz, 1H), 7.16 (s, 2H), 4.59 (d, *J* = 5.5 Hz, 2H), 3.96 (s, 3H), 2.37 (s, 3H).13C NMR (125 MHz, DMSO) δ 165.23, 158.67, 146.49, 138.60, 134.94, 133.22, 131.61, 130.67, 127.88, 126.10, 121.53, 120.32, 112.76, 56.97, 35.37, 21.01. HRMS (ESI) *m*/*z*: [M + H]^+^ calculated for C_18_H_20_N_5_O_4_S 402.1231, found 402.1238.

##### 2.2.7.2. N-((1–(2,4-dimethylphenyl)-1H-1,2,3-triazol-4-yl)methyl)-4-methoxy-3-sulfamoylbenzamide (6x)

White solid, yield: 59%, m.p: 244–246 °C; ^1^H NMR (500 MHz, DMSO) δ 9.14 (t, *J* = 5.6 Hz, 1H), 8.31 (t, *J* = 6.8 Hz, 1H), 8.26 (s, 1H), 8.13 (dd, *J* = 8.7, 2.3 Hz, 1H), 7.33–7.22 (m, 3H), 7.18 (dd, *J* = 7.6, 6.6 Hz, 1H), 7.15 (s, 2H), 4.61 (d, *J* = 5.5 Hz, 2H), 3.96 (s, 3H), 2.36 (s, 3H), 2.11 (s, 3H).13C NMR (125 MHz, DMSO) δ 165.23, 158.66, 145.41, 139.75, 134.46, 133.16 (d, *J* = 12.6 Hz), 132.22, 131.60, 127.84 (d, *J* = 5.5 Hz), 126.17, 125.01, 112.76, 56.97, 35.32, 21.07, 17.82. HRMS (ESI) *m*/*z*: [M + H]^+^ calculated for C_19_H_22_N_5_O_4_S 416.1387, found 416.1395.

##### 2.2.7.3. N-((1–(4-bromophenyl)-1H-1,2,3-triazol-4-yl)methyl)-4-methoxy-3-sulfamoylbenzamide (6y)

White solid, yield: 54%, m.p: 228–230 °C; ^1^H NMR (500 MHz, DMSO) δ 9.18 (t, *J* = 5.5 Hz, 1H), 8.71 (s, 1H), 8.32 (t, *J* = 9.8 Hz, 1H), 8.13 (dd, *J* = 8.7, 2.2 Hz, 1H), 7.89 (t, *J* = 5.8 Hz, 2H), 7.82–7.72 (m, 2H), 7.28 (t, *J* = 11.6 Hz, 1H), 7.16 (s, 2H), 4.60 (d, *J* = 5.5 Hz, 2H), 3.97 (s, 3H). 13C NMR (125 MHz, DMSO) δ 165.24, 158.69, 136.37, 133.22, 131.61, 127.87, 126.06, 122.37, 121.67 (d, *J* = 13.6 Hz), 112.77, 56.98, 35.34. HRMS (ESI) *m*/*z*: [M + 2]^+^ calculated for C_17_H_17_BrN_5_O_4_S 466.0179, found 468.0162.

##### 2.2.7.4. 4-Methoxy-N-((1–(2-methoxyphenyl)-1H-1,2,3-triazol-4-yl)methyl)-3-sulfamoylbenzamide (6z)

White solid, yield: 59%, m.p: 268–270 °C; ^1^H NMR (500 MHz, DMSO) δ 9.16 (t, *J* = 5.6 Hz, 1H), 8.32 (d, *J* = 2.3 Hz, 1H), 8.29 (s, 1H), 8.12 (dt, *J* = 14.1, 7.1 Hz, 1H), 7.60 (dd, *J* = 7.9, 1.6 Hz, 1H), 7.52 (ddd, *J* = 8.5, 7.6, 1.7 Hz, 1H), 7.29 (ddd, *J* = 31.9, 15.8, 5.9 Hz, 2H), 7.15 (s, 2H), 7.12 (dd, *J* = 7.7, 1.1 Hz, 1H), 4.60 (d, *J* = 5.6 Hz, 2H), 3.96 (s, 3H), 3.85 (s, 3H).13C NMR (125 MHz, DMSO) δ 165.25, 158.66, 152.03, 145.25, 133.20, 131.60, 131.05, 130.79, 127.84, 126.28, 126.12, 125.29, 121.36, 113.54, 112.79, 56.96, 56.60, 35.27. HRMS (ESI) *m*/*z*: [M + H]^+^ calculated for C_18_H_20_N_5_O_5_S 418.1180, found 418.1186.

### 2.3. CA inhibition assay

An SX.18 MV-R Applied Photophysics (Oxford, UK) stopped-flow instrument has been used for assaying the CA catalyzed CO_2_ hydration activity^19^. Phenol Red (at a concentration of 0.2 mM) has been used as indicator, working at the absorbance maximum of 557 nm, with 10 mM Hepes (pH 7.4) as buffer, 0.1 M Na_2_SO_4_ or NaClO_4_ (for maintaining constant the ionic strength; these anions are not inhibitory in the used concentration), following the CA-catalyzed CO_2_ hydration reaction for a period of 5–10 s. Saturated CO_2_ solutions in water at 25 °C were used as substrate. Stock solutions of inhibitors were prepared at a concentration of 10 μM (in DMSO-water 1:1, v/v) and dilutions up to 0.01 nM done with the assay buffer mentioned above. At least seven different inhibitor concentrations have been used for measuring the inhibition constant. Inhibitor and enzyme solutions were preincubated together for 10 min at room temperature prior to assay, in order to allow for the formation of the E-I complex. Triplicate experiments were done for each inhibitor concentration, and the values reported throughout the paper are the mean of such results. The inhibition constants were obtained by non-linear least-squares methods using the Cheng-Prusoff equation, as reported earlier^20–24^, and represent the mean from at least three different determinations. All CA isozymes used here were recombinant proteins obtained as reported earlier by our group^10^^c,^^25^^,^^26^.

## 3. Result and discussion

### 3.1. Chemistry

**Scheme 1.**
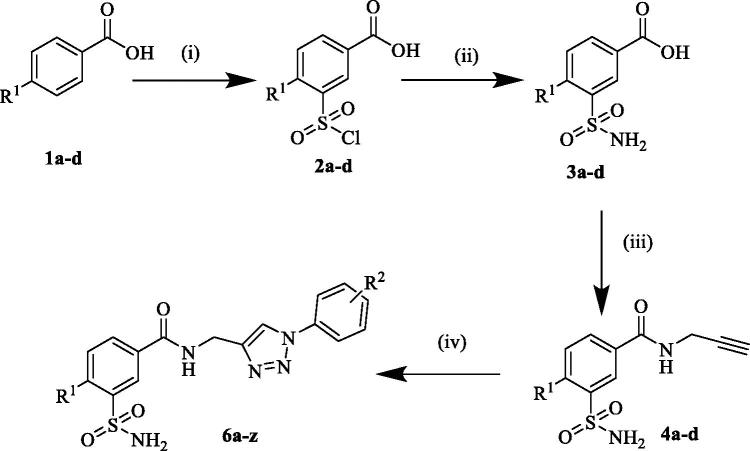
Synthesis of 1,2,3-triazole 3-sulfamoylbenzamide hybrids (**6a–z**). Reagent and reaction conditions**: (**i) HSO_3_Cl, 110 °C, 6–8 h, 70–80%; (ii) NH_4_OH sol., 0 °C, 2 h; (iii) Propargyl amine, EDCI, HOBt, anhydrous DMF, rt, 16–24 h, 74–85%; (iv) substituted phenyl azides, CuSO_4_, Sodium ascorbate, *^t^*BuOH: H_2_O (1:1), 40 °C, 4–6 h, 52–98%^30^.

The present work was aimed at designing molecules, which target specifically CA I, II, IX, XII based on previously reported data. The synthesis of the designed 3-functionalised benzenesulfonamide linked triazoles (**6a–z**) was performed according to the general synthetic route as illustrated in Scheme 1. Commercially available 4-substituted benzoic acid (**1a–d**) were treated with chlorosulfonic acid at 110 °C to afford the 3-(chlorosulfonyl)benzoic acids (**2a–d**), which were treated with ammonium hydroxide solution at 0 °C to get the corresponding 3-sulfamoylbenzoic acids (**3a–d**)^23^.

In the next step, the 3-sulfamoyl benzoic acids **(3a–d**) were coupled with propargyl amine in presence of EDCI and HOBt in the presence of dry DMF to afford the corresponding sulfamoylbenzamide alkyne intermediates (**4a–d**)^24^. In the final step the sulfamoylbenzamide alkyne intermediates (**4a–d**) were subjected to click chemistry reaction with substituted azide intermediates in the presence of CuSO_4_, sodium ascorbate in *t*-BuOH and H_2_O (1:1) solvent system to afford the desired target derivatives (**6a–z**)^13^ in good to excellent yields. The phenyl azide intermediates were previously prepared from the corresponding anilines through diazotization, using concentrated HCl, NaNO_2_, and NaN_3_^27^^,^^28^.

### 3.2. CA inhibition

**Figure 4.**
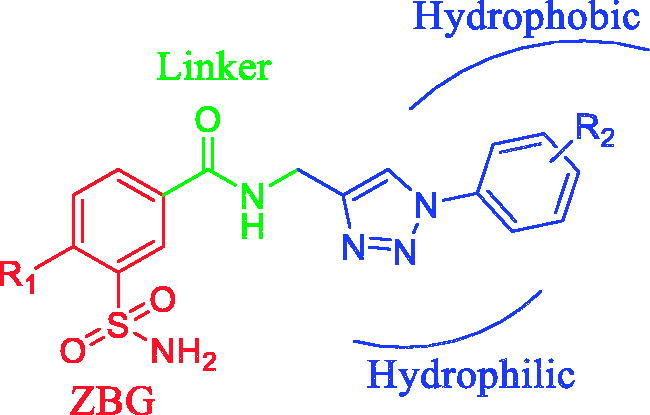
General structure of the synthesised molecules.

The newly synthesised compounds 3-sulfamoylbenzamide linked 1,2,3-triazoles (Figure 4) (**6a–z**) as well as the intermediates (**3a–d**) and (**4a–d**) (listed in Table 1) were screened for their CA inhibitory activities against four physiologically significant isoforms, the cytosolic hCA I (associated with edema) and II (associated with glaucoma) as well as the membrane bound hCA IV (associated with glaucoma and retinitis pigmentosa) and transmembrane hCA IX (associated with tumors) by means of the stopped flow carbon dioxide assays^29^ in comparison to AAZ as standard CAI (Table 2).

**Table 1.** List of synthesised compounds **3a-d, 4a-d** and **6a-z**.

**Table 2.** Inhibition of hCA isoforms I, II, IV and IX with compounds **6a–z**, **3a–d**, **4a–d,** and AAZ as standard inhibitor

K_I_ (nM)*				
Cmp	hCA I	hCAII	hCA IV	hCA IX
6a	752.7	4491	9421.0	7976
6b	854.7	8967	9142	4904
6c	951.9	9271	9272	6924
6d	866.9	2973	8835	7423
6e	430.8	2357	9298	3188
6f	887.9	9012	6673	6746
6g	447.5	366.4	8584	6128
6h	693.0	515.6	1758	225.8
6i	312.1	71.2	170.4	84.4
6j	478.6	51.4	2282	211.9
6k	86.7	56.5	80.0	41.6
6l	50.8	6.5	65.3	64.3
6m	811.2	7.8	89.9	34.6
6n	890.2	49.7	240.0	84.5
6o	69.2	73.5	174.8	63.1
6p	368.9	91.1	91.8	234.6
6q	425.9	328.7	5314	30.8
6r	836.2	260.3	215.3	67.5
6s	696.0	402.2	3397	67.9
6t	696.0	372.1	514.6	71.5
6u	680.8	2898	9349	702.9
6v	571.6	9647	5352	6821
6w	966.8	4158	7621	525.6
6x	7562	6390	9337	93.8
6y	555.1	2329	9082	1873
6z	952.0	5367	8935	768.1
3a	8769	8656	8914	9415
3b	8847	1382	2470	8976
3c	5750	760.0	957.5	9570
3d	9755	9183	8777	9674
4a	9280	513.9	8456	9362
4b	4931	73.5	78.8	446.9
4c	747.2	164.5	429.1	815.9
4d	9501	5531	6843	9221
AAZ	250	12.1	74	25.8

* Mean from three different assays, by a stopped flow technique (errors were in the range of 5–10% of the reported values).

The results of the CA inhibitory assay are discussed below

- The cytosolic isoform hCA I was strongly inhibited by all the synthesised compounds with *K*_I_ ranging between 50.8 nM and 9.755 μM range. Among all the synthesised compounds only three compounds **6k, 6l,** and **6o** were found to be more potent hCA I inhibitors with *K*_I_ 50.8–86.7 nM compared to the standard AAZ (*K*_I_ = 250 nM), in-fact the compound **6k** is almost four fold more active than that of AAZ. The remaining compounds including the intermediates **3a–d** and **4a–d** were showing the weakest inhibition (*K*_I_ ranging between 312.1 and 9755 nM). It was also observed that the 4-chloro and 4-fluoro substituted 3-benzenesulfonamide derivatives were exhibiting the better inhibition as compared to the methoxy derivatives (Table 2).
- The cytosolic isoform hCA II was strongly inhibited by some of the synthesised compounds with *K*_I_ ranging between 6.5 nM and 0.760 μM. Compounds **6l** and **6m** showed excellent inhibition with *K*_I_ 6.5 and 7.8 nM, respectively, compared to the standard AAZ (*K*_I_ = 12.1 nM). The compounds **6a–6f, 6u-6z, 3a, 3b, 4d** were found to be the weakest inhibitors with *K*_I_ 1.382–9.647 μM and the remaining were found weak inhibitors with *K*_I_ < 800 nM. The results show that the 4-chloro substituted 3-benzenesulfonamide derivatives were more potent inhibitors of hCA II (Table 2).
- The membrane bound isoform hCA IV was weakly inhibited by all of the synthesised molecules with *K*_I_ ranging between 78.8 nM and 2.470 μM except one compound **6l,** which was found to be most potent with *K*_I_ 65.3 nM compared to the reference drug AAZ (*K*_I_ = 74 nM). Twelve compounds **6i, 6k-6p, 6r, 6t, 3c, 4b,** and **4c** were showing inhibition with *K*_I_ < 1000 nM. Whereas, the other compounds were showing very weak inhibition with *K*_I_ > 2000 nM (Table 2).
- The tumor associated isoform hCA IX was weakly inhibited by all the synthesised compounds with *K*_I_ ranging between 30.8 nM and 9.674 μM as compared to the standard AAZ (*K*_I_ = 25.8 nM). From the results it is found the the 4-chloro and 4-fluoro substituted 3-benzenesulfonamide derivatives **6i, 6k-6o,** and **6q-6t** were showing moderate inhibition ranging between 30.8 and 84.5 nM (Table 2).

Surprisingly, the structure activity relationship (SAR) studies revealed that the derivatives containing –Cl, –F substitution on the C-4 position of 3-sulfamoylbenzamide moiety (**6i–6t**) were exhibiting strong inhibition against all four isoforms hCA I, II, IV, and IX, whereas all the intermediates i.e. the benzoic acid derivatives **3a–3d** and N-propargyl benzamide **4a–4d** derivatives showed very poor inhibition, except **4b,** which showed moderate inhibition against hCA II and IV with *K*_I_ 0.073 and 0.078 μM, respectively. The compound **6 l** showed excellent inhibition against hCA I, II, IV as compared to AAZ the standard drug used in these assays.

It was also observed from SAR studies that the substitution on the phenyl ring of 1,2,3-triazole moiety led to a dramatic change of inhibition potency. The 4-substituted –F, –CF_3_, –OMe (**6k, 6l, 6m, and 6o**) showed strong inhibition against hCA I, II, IV. Hence, in a broader sense the compounds were excellent inhibitors if the 3-sulfamoyl phenyl ring was substituted with electron withdrawing groups. Furthermore, while comparing with the 4-functionalised benzenesulfonamides, the 3-functionalised benzenesulfonamides were more selective towards hCA I, II, IV than towards hCA IX.

## 4. Conclusions

In conclusion, a series of new 1,2,3-triazole derivatives containing primary 3-benzenesulfonamide moiety (**6a–6z**) were synthesised using click tailing approach to develop CA inhibitors via covalent linking to the selected fragment onto the CA pharmacophore. All the synthesised compounds were assayed as CA inhibitors against pharmacologically relevant isoforms i.e. cytosolic isoforms hCA I, II; membrane bound isoform hCA IV and transmembrane isoform hCA IX. The synthesised compounds showed inhibition in low to medium nanomolar range. The newly synthesised compounds showed weak inhibitory potency against hCA IX whereas compound **6l** showed excellent inhibition against hCA I, II, and IV with K_I_ values of 50.8, 6.5, and 65.3 nM with compared to AAZ as standard drug with *K*_I_ = 250, 12.1, 74 nM, respectively. Furthermore, the results of hCA inhibition clearly indicate that few of the compounds containing electron withdrawing substitution on both the phenyl rings (**6k, 6l, 6m,** and **6o**) showed strong inhibitory activity against three isoforms hCA I, II, IV. Hence, it may be concluded that the newly synthesised 1,2,3-triazole 3-sulfamoylbenzamide derivatives exhibit potent hCA inhibitory properties.
